# Multiple *Streptococcus sanguinis* brain abscesses misdiagnosed as cerebral manifestation of tuberculosis

**DOI:** 10.1590/0037-8682-0391-2022

**Published:** 2022-12-16

**Authors:** Ismet Mirac Cakir, Tumay Bekci, Uluhan Eryuruk

**Affiliations:** 1 Giresun University, Faculty of Medicine, Department of Radiology, Giresun, Turkey.

**FIGURE 1:**
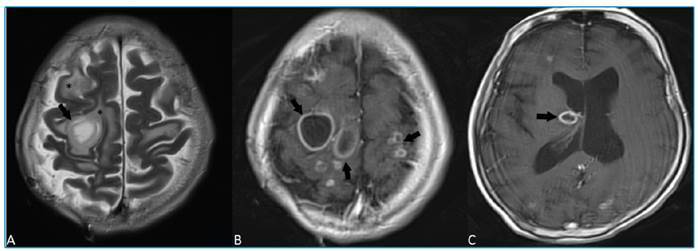
**(A)** Axial T2-weighted images showing hyperintense lesion (arrow) with surrounding edema (asterisk) of right frontal lobe. **(B-C)** Axial post contrast T1-weighted images showing ring-enhancing lesions (arrows) of bilateral parietal and frontal lobes, and right thalamus.

**FIGURE 2:**
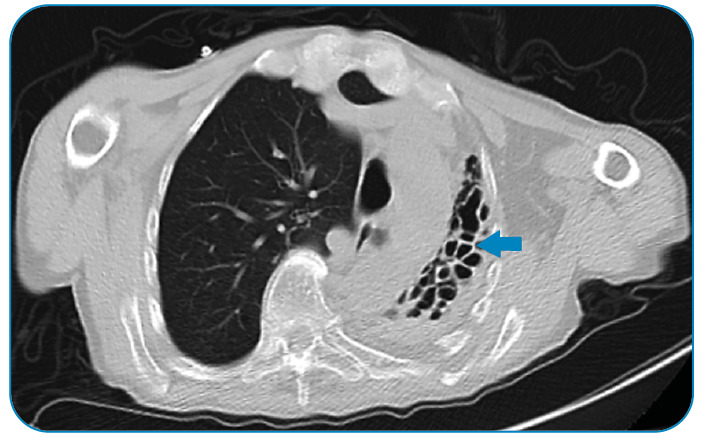
Thorax computed tomography showing unilateral tuberculous lung destruction **(arrow)**.

A 68-year-old man with a known history of pulmonary tuberculosis (TB) was admitted to our hospital following sudden onset of left hemiplegia. Cranial magnetic resonance imaging (MRI) showed multiple hyperintense lesions on T2-weighted images with surrounding vasogenic edema (Figure 1A) and ring-enhancement following administration of an intravenous contrast agent (Figure 1B-C). MR spectroscopy revealed lipid and lactate peaks with low levels of n-acetyl aspartate, consistent with the inflammatory process. There were no abnormal findings, except the TB-induced left lung damage on thoracic computed tomography (Figure 2); infective endocarditis was not evident on transesophageal echocardiography. The patient was preliminarily diagnosed with TB abscess due to a history of pulmonary TB and was administered anti-tubercular treatment. As the cranial lesions progressed under TB treatment, surgical drainage was performed, and pus culture from the abscess showed *Streptococcus sanguinis.*

*S. sanguinis* is a facultative anaerobic commensal bacterium found in the oral cavity. Central nervous system infectiondue to *S. sanguinis* has rarely been reported in the literature. Facilitating conditions included infective endocarditis, pulmonary arteriovenous fistulas, and history of craniotomy in previously reported cases^1^ ^-^ ^3^; however, neither foci of infection nor facilitating causes were found in the present case. 

To our knowledge, this is the most severe case of brain abscess caused by *S. sanguinis*. Moreover, the diagnostic challenge posed by the patient’s history of pulmonary TB is noteworthy. Awareness of this pathogen as a rare cause of brain abscess will help clinicians determine the accurate diagnosis.
